# Exosomal miR-2137 from cadmium-treated hepatocytes drives renal ferroptosis via GPX4 suppression and is alleviated by selenium

**DOI:** 10.3389/fcell.2025.1585106

**Published:** 2025-07-30

**Authors:** Qian Wen, Meiyan Qi, Yanjun Wu, Weiwei Ji, Lixing Zhan

**Affiliations:** Shanghai Institute of Nutrition and Health, University of Chinese Academy of Sciences, Chinese Academy of Sciences, Shanghai, China

**Keywords:** cadmium, ferroptosis, GPx4, hepatorenal communication, selenium

## Abstract

Cadmium (Cd) is a toxic heavy metal that primarily affects the liver and kidneys. Despite greater Cd accumulation in the liver, Cd-induced oxidative damage is more pronounced in the kidney, suggesting the involvement of hepatorenal communication. However, the underlying mechanism remains unclear. To investigate Cd-induced hepatorenal toxicity, we established a Cd-exposed mouse model and assessed ferroptosis-related liver and kidney injury. Exosomes derived from Cd-exposed hepatocytes were isolated, and miRNAs targeting GPX4 were screened and identified. The role of GPX4-targeting miRNAs in mediating renal toxicity induced by hepatocyte-derived exosomes was evaluated *in vivo* using antagomirs. The protective effect of selenium (Se) supplementation against Cd-induced hepatic and renal damage was also examined. Cd exposure induced significant liver and kidney injury through GPX4-downregulated ferroptosis. Mechanistically, exosomes from Cd-treated hepatocytes were enriched in miR-2137, which targets renal GPX4 and promotes ferroptosis in the kidney. Injection of hepatocyte-derived exosomes alone reduced renal GPX4 levels *in vivo*, an effect that was reversed by miR-2137 antagomir treatment. Furthermore, Se supplementation restored GPX4 expression and protected both liver and kidney tissues from Cd-induced damage. These findings reveal a novel exosome-mediated hepatorenal communication pathway under Cd exposure, wherein hepatocyte-derived exosomal miRNAs contribute to distant renal injury. Targeting specific exosomal miRNAs or enhancing GPX4 expression via selenium may offer therapeutic strategies against Cd toxicity.

## 1 Introduction

Cadmium (Cd) is a toxic metal for the organism. Cd pollution has become a global environmental issue due to the ease with which Cd accumulates in ecosystems, the food chain and human bodies (Järup, 2003; Nordberg, 2009; Wei et al., 2023). Cd is toxic to a number of organs and systems, including the liver, kidneys, bones, hematopoietic system, respiratory system, and reproductive system (Friberg, 1983). This can ultimately result in a number of health issues, including osteoporosis, diabetes mellitus, hyperlipidemia, cardiovascular disease, and various tumors (Satarug et al., 2010; Fagerberg and Barregard, 2021; Hong et al., 2021). The accumulation of Cd in tissues has a significant impact on cellular functions, including the generation of reactive oxygen species (ROS), which causes oxidative stress, induces cell damage and apoptosis, interferes with the DNA repair system, destroys the cellular antioxidant defense system, and ultimately leads to tissue damage (Ma et al., 2022; Peana et al., 2022; Yi et al., 2022). The liver and kidneys are the primary organs responsible for storing Cd within the body. Approximately half of the accumulated Cd is stored in these organs (Friberg, 1983; Satarug et al., 2010). Upon entering the human body, Cd initially forms complexes with metallothionein and accumulates in the liver. These Cd–metallothionein complexes are subsequently transported via the bloodstream and reabsorbed by the kidneys. Within renal lysosomes, the complexes are degraded, leading to the release of free Cd ions. This disrupts lysosomal homeostasis and results in the release of hydrolytic enzymes, ultimately causing cellular injury (Nordberg, 1984; Nordberg and Nordberg, 2022). Cd^2+^ can enter mitochondria, where they interfere with the electron transport chain (ETC.) and oxidative phosphorylation. This disruption leads to the accumulation of ROS, mitochondrial dysfunction, and may ultimately trigger apoptosis (Lee et al., 2004; Gobe and Crane, 2010). In addition, Cd disrupts endoplasmic reticulum (ER) calcium homeostasis, thereby activating the unfolded protein response (UPR) and pro-apoptotic signaling pathways (Biagioli et al., 2008; Le et al., 2016). Previous studies mainly focused on the direct toxicity of Cd to the liver or kidney, physiologically. In hepatorenal syndrome, hepatic dysfunction leads to progressive renal failure, highlighting a high degree of physiological crosstalk between the liver and kidneys (Simonetto et al., 2020). These observations have prompted growing interest in the mechanisms of inter-organ communication under Cd exposure. Nevertheless, the extent to which Cd exerts systemic toxicity through the coordinated dysfunction of the liver and kidney remains poorly understood, and has been insufficiently explored in the current literature.

Exosomes represent one of the principal pathways facilitating inter-organ communication, and thus play an integral role in regulating organ communication within the body (Kita et al., 2019). Exosomes are vesicles with a diameter of approximately 40 nm to 1 μm, secreted by all cells (Kalluri and LeBleu, 2020). They originate from the plasma membrane or endosomes (Bebelman et al., 2018). A multitude of active cellular components, including DNA, RNA, lipids, metabolites, and cytoplasmic and cell surface proteins, can be transported in exosomes to target cells, where they function (Kalluri, 2016; Mathieu et al., 2019). As a pivotal mediator of organ communication, exosomes serve as a crucial conduit for interorgan communication between the liver and the kidney (Gao et al., 2020). Hence, we proposed that the communication between liver and kidney mediated by exosomes may be involved in Cd damage.

Ferroptosis is defined as a form of cell death that is dependent on iron and involves lipid peroxidation (Wang et al., 2023). A number of pathways have been demonstrated to be resistant to ferroptosis, with the GPX4-regulated pathway representing the most well-characterized example. GPX4 relies on glutathione (GSH) to prevent ferroptosis by reducing phospholipid peroxides to the corresponding phosphatidyl alcohols (Jiang et al., 2021). Cd-induced oxidative stress may be the reason to trigger ferroptosis and thus the manipulation to restore GPX4 expression represents an effective therapeutic strategy to diminish the oxidative stress for Cd toxic rescue.

GPX4 is a selenoenzyme that reduces complex hydroperoxides in order to maintain cellular redox homeostasis, utilizing GSH as a cofactor (Xu et al., 2021). The selenoproteins contain at least one selenocysteine (Sec), which is essential for their oxidoreductase function. Selenium (Se) is therefore an essential mineral that can function by being inserted into selenoproteins in the form of selenocysteines (Kryukov et al., 2003; Rayman, 2012). Consequently, the administration of Se supplements facilitates the biosynthesis of selenocysteine and selenoproteins, including the upregulation of GPX4 expression. Nevertheless, the precise mechanism through which Se exerts its effects on Cd-induced tissue ferroptosis remains unclear. It is therefore essential to pursue further research into the mechanism by which Se antagonizes Cd-induced toxicity in order to gain new insights into this process. Our findings indicate the potential utility of exosomes as biomarkers and therapeutic agents for the treatment of Cd toxicity. In this study, by constructing a mouse model of Cd exposure and Se supplementation, we want to reveal the role and mechanism of organ communication in Cd exposure leading to organism toxicity, and provide a theoretical basis for the toxicology of Cd as a heavy metal.

## 2 Materials and methods

### 2.1 Chemicals and reagents

The chemicals CdCl_2_ (No. 202908) and L-SeMet (No. 561505) were procured from Sigma-Aldrich (St. Louis, MO, United States). Liproxstatin-1 (No: 950455-15-9) was procured from MedChem Express (Monmouth Junction, NJ, United States). The antibodies against GPX4 (No: A11243) and FTH1 (No: 19544) were obtained from ABclonal (Wuhan, China). The Cell Counting Kit-8 (CCK-8, No. MA0218) was procured from MeilunBio (Dalian, China).

The AST and ALT kits for liver function detection were procured from Shensuoyoufu (Shanghai, China), while the AKP detection kit (No: A059-1-1) was obtained from the Nanjing Jiancheng Bioengineering Institute (Nanjing, China). The kits for the detection of blood urea nitrogen (BUN, No: C013-1-1), creatinine (CRE, No: C011-2-1), and uric acid (UA, No: C012-1-1) for the assessment of renal function were obtained from the Nanjing Jiancheng Bioengineering Institute (Nanjing, China). The malondialdehyde (MDA, No: S0131) assay kit and GSH and GSSG assay kit (No: S0053) were obtained from Beyotime (Shanghai, China). The BODIPY™ 581/591 C11 fluorescence stainer (No: D3861) was obtained from Thermo Fisher Scientific (Waltham, MA, United States). The ROS assay kit (No: S0033) was obtained from Beyotime (Shanghai, China). The myeloperoxidase (MPO) assay kit (No: A044-1-1) was sourced from the Nanjing Jiancheng Bioengineering Institute (Nanjing, China).

### 2.2 Animals and treatment

The wild-type C57BL/6J mice were procured from the Shanghai Laboratory Animal Co. (Shanghai, China). All mice were male, aged between 8 and 12 weeks, and weighed between 20 and 30 g. Mice were maintained on a diet supplemented with 2 ppm SeMet for 3 months. This concentration was selected based on previous studies demonstrating its safety and efficacy in murine models (Rodríguez-Sosa et al., 2013). During the final 7 days, mice received daily intraperitoneal injections of CdCl_2_ (1.25 mg/kg). Tissues were collected at the end of the treatment period for subsequent analyses.

### 2.3 Cell lines

The human liver hepatocellular cell line Huh7, the kidney cell line HK2 and the 293T cell were cultured in Dulbecco’s Modified Eagle’s Medium (DMEM) (high sugar, containing 10% fetal bovine serum and 1% penicillin-streptomycin double antibody solution) (GIBCO, Thermo Fisher Scientific). AML12 was cultured in DMEM/F-12 (Dulbecco’s Modified Eagle Medium/Nutrient Mixture F-12) (GIBCO, Thermo Fisher Scientific). The medium was changed every two or 3 days, and the cells were digested and passaged using trypsin (0.25%) (GIBCO, Thermo Fisher Scientific). All cells were incubated at 37°C in a humidified atmosphere containing 5% CO_2_ in order to maintain optimal conditions for cell growth.

### 2.4 Liver primary cell isolation

Following anaesthesia of the mice, primary hepatocytes were isolated by collagenase perfusion and an intravenous indwelling needle was inserted and fixed in the superior vena cava. Subsequently, the inferior vena cava was incised, and Krebs Ringer’s buffer (2 mL/min) containing glucose was injected to clear the liver blood. Subsequently, the liver was perfused with a digestion solution comprising 800 U of collagenase I (Worthington) in Krebs Ringer’s buffer with dextrose for a period of 20 min at a flow rate of 2.5 mL/min. Following digestion, the entire liver was excised and placed in pre-cooled Dulbecco’s Modified Eagle Medium (DMEM) and maintained on ice for subsequent isolation and dissociation (within 2 hours of collection). The resulting hepatocyte suspension was subjected to centrifugation (50 g, 4°C, 10 min) on 45% Percoll (Sigma-Aldrich) in order to separate live and dead cells. Following the recovery of the live cells, they were resuspended in DMEM supplemented with 10% fetal bovine serum (Hyclone) and 2% penicillin/streptomycin. The cells were then distributed across cell culture plates and grown at 37°C, 5% CO_2_ for the duration of the experiment.

### 2.5 Protein extraction and immunoblotting

Total protein lysates were homogenized in RIPA buffer plus protease inhibitors (Vanadate, Aprotinin, Leupeptin, Pepstain A, PMSF) and centrifuged with 12,000 rpm for 10 min at 4°C. Protein concentration was measured by SpectraMax M5 (SoftMax Pro6) using the Pierce BCA Assay Kit (ThermoFisher Scientific). Supernatant was used for immunoblotting with the indicated antibodies. Signal was detected using chemiluminescent HRP Substrate (share-bio) and chemiluminescence apparatus (Tanon, China).

### 2.6 RNA extraction and quantitative real-time PCR (qRT-PCR)

Total RNA was extracted from cells and tissues using TRIzol reagent (Invitrogen), following the manufacturer’s instructions. For mRNA quantification, 1 μg of RNA was reverse-transcribed using the PrimeScript™ RT Reagent Kit (Takara, RR037A) according to the manufacturer’s protocol. For miRNA analysis, approximately 100 ng of total RNA was reverse-transcribed using M-MLV reverse transcriptase (Promega, M1705) with specific stem–loop primers for miRNAs and a reverse primer for U6 (Table 1). The conditions for reverse transcription of mRNA are as follows: 37°C for 15 min, followed by 85°C for 5 s. The reverse transcription reaction of miRNA was carried out at 42°C for 1 h.

**TABLE 1:** Primers for miRNAs and U6 revserse transcription.

Gene	Primer (5′-3′)
miR-15a-5p RT	CTCAACTGGTGTCGTGGAGTCGGCAATTCAGTTGAGCACAAACC
miR-383-3p RT	CTCAACTGGTGTCGTGGAGTCGGCAATTCAGTTGAGTCTGACCA
miR-2137 RT	CTCAACTGGTGTCGTGGAGTCGGCAATTCAGTTGAG CTCCCTGG
miR-30a-3p RT	CTCAACTGGTGTCGTGGAGTCGGCAATTCAGTTGAG GCTGCAAA
miR-205-5p RT	CTCAACTGGTGTCGTGGAGTCGGCAATTCAGTTGAG CAGACTCC
miR-700-5p RT	CTCAACTGGTGTCGTGGAGTCGGCAATTCAGTTGAG GCAAGCAC
miR-346-3p RT	CTCAACTGGTGTCGTGGAGTCGGCAATTCAGTTGAG GCT GCA GG
miR-140-3p RT	CTCAACTGGTGTCGTGGAGTCGGCAATTCAGTTGAG GTCCGTGG
U6-RT	ATA TGGAACGCTT CACG

The resulting cDNA was diluted 100-fold and subjected to quantitative real-time PCR using Hieff UNICON® Power qPCR SYBR Green Master Mix (Yeasen, 11196ES03) on a QuantStudio 6 Real-Time PCR System. Primer sequences used for mRNA and miRNA detection are listed in Table 2, respectively. Relative mRNA or miRNA expression was calculated using the ΔΔCt method, with normalization to GAPDH for mRNAs, and U6 snRNA for miRNAs.

**TABLE 2:** Primers for quantitative real-time PCR (qRT-PCR).

Gene	Primer (5′-3′)
GAPDH-F	AGGTCGGTGTGAACGGATTTG
GAPDH-R	GGGGTCGTTGATGGCAACA
GPX4-F	TGTGCATCCCGCGATGATT
GPX4-R	CCCTGTACTTATCCAGGCAGA
FTH1-F	CAAGTGCGCCAGAACTACCA
FTH1-R	ACAGATAGACGTAGGAGGCATAC
SLC3A2-F	GACACCGAAGTGGACATGAAA
SLC3A2-R	GCTCCTCCTTGGATAAGCCG
SLC7A11-F	GGCACCGTCATCGGATCAG
SLC7A11-R	CTCCACAGGCAGACCAGAAAA
SLC40A1-F	TGGAACTCTATGGAAACAGCCT
SLC40A1-R	TGGCATTCTTATCCACCCAGT
shGPX4-F1	CCGGGTGAGGCAAGACCGAAGTAAACTCGAGTTTACTTCGGTCTTGCCTCACTTTTTG
shGPX4-R1	AATTCAAAAAGTGAGGCAAGACCGAAGTAAACTCGAGTTTACTTCGGTCTTGCCTCAC
shGPX4-F2	CCGGGTGGATGAAGATCCAACCCAACTCGAGTTGGGTTGGATCTTCATCCACTTTTTG
shGPX4-R2	AATTCAAAAAGTGGATGAAGATCCAACCCAACTCGAGTTGGGTTGGATCTTCATCCAC
miR-15a-5p F	ACACTCCAGCTGGGTAGCAGCACATAATGG
miR-383-3p F	ACACTCCAGCTGGGCCACAGCACTGCCTG
miR-2137 F	ACACTCCAGCTGGGGCCGGCGGGAGCCCC
miR-30a-3p F	ACACTCCAGCTGGGCTTTCAGTCGGATGTT
miR-205-5p F	ACACTCCAGCTGGGTCCTTCATTCCACCGG
miR-700-5p F	ACACTCCAGCTGGGTAAGGCTCCTTCCTGT
miR-346-3p F	ACACTCCAGCTGGGAGGCAGGGGCTGGGCC
miR-140-3p F	ACACTCCAGCTGGGACCACAGGGTAGAACC
Universal-R	TCAACTGGTGTCGTGGAGTCG
U6-F	CTCGCTTCGGCAGCACA
U6-R	ATATGGAACGCTTCACG

### 2.7 Mass spectrometry (MS)

The objective of this study is to determine the concentration of Se and Cd in biological tissues and body fluids using inductively coupled plasma mass spectrometry (ICP-MS). A total of 200 μL of whole blood and 200 mg of tissue were subjected to mass spectrometry pre-treatment through the addition of concentrated nitric acid (67%-70% concentration) for microwave digestion, with the objective of subsequent mass spectrometry detection.

### 2.8 Histological analyses

The liver and kidney tissues were fixed with 4% paraformaldehyde for a period of 24 h, following which they were dehydrated with an ethanol gradient and subsequently paraffin embedded. The tissues were then sectioned and stained with hematoxylin and eosin. The histopathological alterations were examined under a light microscope (ECHO).

### 2.9 Cell survival assay

HK2 cells and AML12 cells were inoculated in 96-well plates at appropriate cell densities, in accordance with the experimental design. At the conclusion of the drug treatment period, the plates were washed with PBS, and 10 μL of CCK-8 solution was added to each well. The cell viability was then determined at 450 nm after incubation for 1 h in the incubator.

### 2.10 Lipid peroxidation and ROS measurement

To ascertain the extent of cellular ferroptosis, the cells were distributed in six-well plates and, upon completion of the drug treatment, the cells were subjected to digestion and collection. The BODIPY 581/591 C11 staining working solution or DCFH-DA probe was then added to the medium, incubated in the incubator, and subsequently washed. The fluorescence intensity was then detected using flow cytometry.

### 2.11 GSH/GSSG assay

A total of 10 mg of tissue was obtained and the levels of GSH and GSSG were quantified following dilution in accordance with the manufacturer’s instructions for the GSH and GSSG kit.

### 2.12 RNA-seq and data processing

Total RNA was extracted from isolated liver tissues using the TRIzol method. The RNA samples and cDNA libraries were constructed and sequenced by Majorbio Biotech. Majorbio Biotech conducted differential gene analysis and enrichment analysis.

### 2.13 Exosome extraction

Exosomes were separated from blood and cell supernatants using differential centrifugation. For serum samples, centrifugation was conducted at 2,500 g for 5 min at 4°C, and the supernatant was obtained after filtration at 0.45 μm and subsequent centrifugation at 13,200 g for 22 min at 4°C. Subsequently, filtration through a 0.22 μm filter was conducted on two occasions, followed by centrifugation at 120,000 g for a period of 2 h at 4°C. Once the centrifugation process was complete, the resulting precipitate was transferred for subsequent processing.

The Huh7 cell line was selected for extracting exosomes from cell supernatants because of its high exosome production. The cell supernatant should be subjected to centrifugation at 800 *g* for a period of 10 min at 4°C. Following this, the supernatant should be transferred to a new centrifuge tube and subjected to centrifugation at 20,000 g for a period of 20 min at 4°C. Subsequently, a 0.22 μm filtration was conducted, followed by centrifugation at 100,000 g for a period of 2 h at 4°C. Once the centrifugation process was complete, the precipitate was removed for subsequent processing, while a portion of the assay protein concentration was employed for exosome quantification.

### 2.14 Mouse organoid generation

Following isolation of primary hepatocytes from mouse liver, 4,000 cells were resuspended in 50 μL of cold Matrigel (Corning, 356231) and seeded into 24-well plates. The plates were incubated at 37°C for 10 min to allow Matrigel polymerization. Subsequently, pre-warmed Hep-Medium was added to each well, supplemented with 5 μM SeMet and 100 nM CdCl_2_. On day 12, bright-field images were captured, and organoid diameters were quantified using ImageJ software.

Hep-Medium was prepared using Advanced DMEM/F12 (Thermo Fisher Scientific) supplemented with 15% RSPO1-conditioned medium (homemade), B27 supplement minus vitamin A (Thermo Fisher Scientific), 50 ng/mL epidermal growth factor (EGF, BioLegend), 1.25 mM N-acetylcysteine (Sigma-Aldrich), 10 nM gastrin (Sigma-Aldrich), 3 μM CHIR99021 (Sigma-Aldrich), 25 ng/mL hepatocyte growth factor (HGF, BioLegend), 50 ng/mL fibroblast growth factor 7 (FGF7, BioLegend), 50 ng/mL fibroblast growth factor 10 (FGF10, BioLegend), 1 μM A83-01 (Sigma-Aldrich), 10 mM nicotinamide (Sigma-Aldrich), and 10 μM Rho-associated kinase (ROCK) inhibitor Y-27632 (Calbiochem). The medium was freshly prepared or stored at 4 °C for up to 1 week before use. Medium was changed every other day during culture.

### 2.15 Transmission electron microscopy

AML12 cells were seeded into 10 cm culture dishes and pre-treated with 15 μM selenomethionine (SeMet) for 48 h, followed by treatment with 70 μM CdCl_2_ for 24 h. Cells, along with the culture medium, were collected into conical centrifuge tubes and centrifuged at 2,000 rpm for 5 min. After discarding the supernatant, the cell pellet was gently resuspended in 2.5% glutaraldehyde (prepared in 0.1 M PBS, pH 7.4) using a pipette, and fixed at 4°C for 4 h, followed by storage overnight at 4°C.

After fixation, cells were washed three times with PBS for 10 min each. Post-fixation was performed in 1% osmium tetroxide (OsO_4_) for 1 h at room temperature, followed by two additional PBS washes. Samples were then dehydrated through a graded ethanol series (30%, 50%, 70%, 80%, 90%, 95%, and 100%), with each step lasting 10–15 min. The 100% ethanol step was repeated twice. Ethanol was subsequently replaced with acetone for intermediate treatment, 10 min per step. Dehydrated samples were infiltrated with a 1:1 mixture of Epon or Spurr resin and acetone for 2 h, followed by pure resin infiltration overnight. Polymerization was carried out at 60°C for 48 h.

The embedded blocks were sectioned into ultrathin slices (∼70 nm) using an Ultracut UCT ultramicrotome (Leica), and the sections were mounted on copper grids. Sections were stained with 2% uranyl acetate for 15 min and then with 0.4% lead citrate for 10 min, with triple rinses in distilled water after each staining step to remove background residues. Finally, the samples were examined under a JEOL 1230 transmission electron microscope (TEM) at an accelerating voltage of 80 kV.

### 2.16 Statistical analysis

All experimental data are presented as mean ± SEM as stated from at least three independent experiments. Student’s t-test between two groups and two-way ANOVA across multiple groups were used to calculate P-values by GraphPad Prism 8. Statistical significance is displayed as *p < 0.05, **p < 0.01, ***p < 0.001, or not significant (ns: p ≥ 0.05).

## 3 Results

### 3.1 Liver and kidney damage under the treatment of Cd is rescued by Se

**FIGURE 1:**
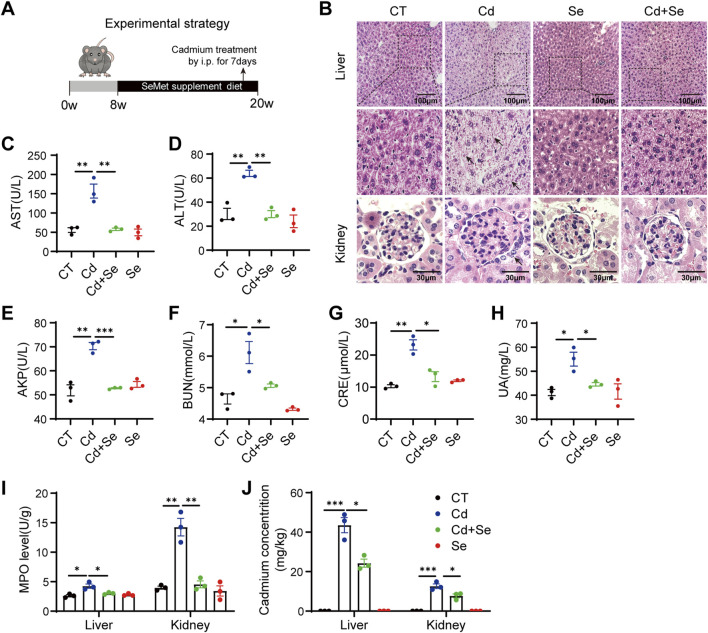
Cd induces liver and kidney damage and is rescued by Se. **(A)** Experimental strategy: 8w mice were given SeMet-supplemented diet for 3months, and CdCl_2_ was administered intraperitoneally at the same time for the last 7 days **(B)** HE staining of pathological sections of mouse liver and kidney. The black arrows indicate damaged hepatocytes and renal tubular cells. Liver scale: 100 μm, Kidney scale: 30 μm. **(C–E)** Mouse Liver Function Test: The kits detect mouse serum AST, ALT and AKP levels. **(F–H)** Mouse Kidney Function Test: The kits detect serum BUN, CRE and UA levels in mice. **(I)** Myeloperoxidase (MPO) levels were assayed in the liver and kidney of differently treated mice. **(J)** Mass spectrometric detection of cadmium accumulation in liver and kidney of mice under different treatments. Symbol colors represent treatment groups: black = control, blue = Cd, green = Cd + Se, and red = Se. Data are expressed as mean ± SEM. Pairwise comparisons between groups were conducted using two-tailed non-paired Student’s t tests. ns, not significant (p ≥ 0.05); *p < 0.05; **p < 0.01; ***p < 0.001.

In order to measure the crosstalk between the liver and the kidney in the organismal toxicity of Cd and the role of Se supplementation in Cd poisoning in mice, a specified mouse model was first constructed. The mice were fed diets supplemented with Se for a period of 3 months, with selenomethionine (SeMet) added to the grain at a final concentration of 2 ppm. Over the final 7 days, the mice were administered CdCl_2_ intraperitoneally on a daily basis in order to expose them to Cd (Figure 1A). The primary targets affected by Cd toxicity are the liver and kidneys (Satarug, 2023). Consequently, our investigation was primarily focused on examining the extent of liver and kidney injury. Histological examination revealed that Cd treatment resulted in histopathological damage to the liver and evident kidney injury, as evidenced by the necrosis and inflammation of hepatocytes, vacuolization of renal tubular epithelial cells and necrosis of glomerular cells (Figure 1B). These effects were markedly attenuated by Se supplementation, as compared to the control group (Figure 1B). No notable alterations were discerned in the Se-treated cohort (Figure 1B). The biochemical indexes of mouse serum demonstrated that the indicators of liver injury, aspartate aminotransferase (AST), alanine aminotransferase (ALT) and alkaline phosphatase (AKP), exhibited a notable elevation following Cd exposure (Figures 1C–E). Similarly, BUN, CRE and UA, which indicate renal function, were also significantly elevated (Figures 1F–H). However, Se supplementation reduced the extent of Cd-induced hepatic and renal injury (Figures 1C–H). Therefore, Se supplementation was found to be an effective method of mitigating the adverse effects of Cd exposure on the liver and kidneys of mice.

In order to assess the extent of damage to liver and kidney tissue, we conducted an examination of myeloperoxidase (MPO), which serves as an indicator of both inflammation and oxidative stress. The administration of Cd resulted in a notable elevation in the levels of MPO in both the liver and kidney tissues. It was unexpected that the level of MPO in kidney tissues was three times higher than that of liver MPO, indicating that the kidney is more severely damaged than the liver in the case of Cd exposure (Figure 1I). Of interest, through mass spectrometry, we observed a higher accumulation of Cd in the liver than in the kidney, yet found that Cd exposure induced a more severe oxidative damage in kidney (Figure 1J). This finding indicates that the kidney is particularly susceptible to Cd treatment. It is also conceivable that, similar to the hepatorenal syndrome, there may be some degree of crosstalk between the liver and the kidney, whereby the liver-derived medium may result in even worse damage to the kidney.

### 3.2 Ferroptosis contributes to Cd-induced liver damage

**FIGURE 2:**
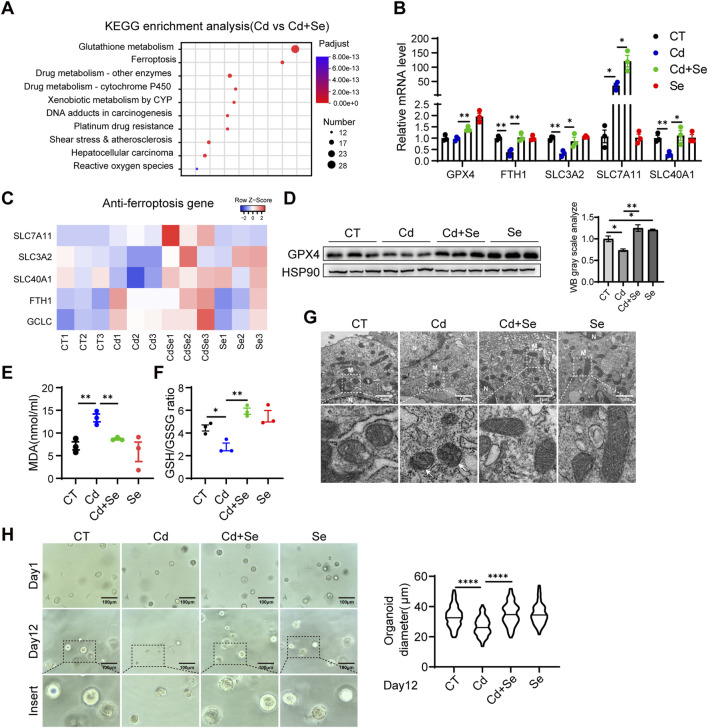
Ferroptosis contributes to Cd-induced liver damage. **(A)** Liver KEGG enrichment analysis and **(B)** Heat map of anti-ferroptosis genes in mice in the cadmium-treated and selenium-cadmium co-treated groups. **(C)** Expression of anti-ferroptosis genes in mouse liver by reverse transcription-polymerase chain reaction (RT-PCR). Values were normalized to the mRNA level of the housekeeping gene Actin in the respective sample. **(D)** Immunoblotting of GPX4 and Hsp90 in mouse liver. The right panel shows the quantitative analysis of GPX4 protein expression. **(E)** Malondialdehyde (MDA) levels and **(F)** GSH/GSSG levels were detected in the livers of mice. **(G)** Transmission electron microscopy of mitochondrial ferroptosis in AML12 cells. Representative mitochondria are shown in white boxes. Scale: 1 μm. N. nucleus; M. mitochondria. The white arrows indicate mitochondria exhibiting typical morphological features of ferroptosis, including reduced mitochondrial volume, increased membrane density, and decreased or vanished cristae structures. **(H)** Representative images of mouse liver organoids treated with 1.25 μM CdCl2 and 10 μM SeMet alone or in combination for 12 days. Statistical graph of class organ diameters (right panel). Scale: 100 μm. Data are expressed as mean ± SEM. Pairwise comparisons between groups were conducted using two-tailed non-paired Student’s t tests. ns, not significant (p ≥ 0.05); *p < 0.05; **p < 0.01; ***p < 0.001.

We hypothesized that Cd caused crosstalk between the liver and kidney, and in order to explore the main damage patterns that occur in the liver of mice after Cd treatment, we conducted an RNA-seq analysis of liver tissues. A comparison of the differentially expressed genes between the Cd-treated group and the Se-Cd combination-treated group revealed significant alterations in the ferroptosis pathway, as demonstrated by the KEGG (Kyoto Encyclopedia of Genes and Genomes) enrichment analysis (Figure 2A). Our findings indicated that the ferroptosis-associated genes, which are essentially ferroptosis-resistant genes, were significantly downregulated in the Cd-treated group. This effect was blunted by Se co-treatment (Figure 2B), which suggests that ferroptosis may be the primary mechanism underlying Cd-induced liver injury. This is characterized by the disruption of the cellular ferroptosis-protective mechanism. It is of particular significance that this destruction can be rectified through the administration of Se. The genes implicated in ferroptosis resistance include solute carrier family 3 member 2 (SLC3A2), solute carrier family seven member 11 (SLC7A11), solute carrier family 40 member 1 (SLC40A1), ferritin heavy chain 1 (FTH1) and glutamate–cysteine ligase catalytic subunit (GCLC) (Figure 2B). The downregulation of SLC40A1 and FTH1 impairs iron export and storage, respectively, leading to intracellular iron accumulation, which in turn promotes Fenton reactions, lipid peroxidation, and ROS generation. Meanwhile, reduced expression of SLC3A2 and GCLC disrupts cystine uptake and glutathione synthesis, weakening the antioxidant defense system and impairing GPX4 activity. These changes collectively lead to the loss of redox homeostasis and the initiation of ferroptosis. The heterodimer of system xc-, comprising the light chain subunit SLC7A11 and the heavy chain subunit SLC3A2, is responsible for the cellular uptake of cystine in exchange for intracellular glutamate (Liu et al., 2020). SLC40A1 plays a crucial role in the transportation of iron from intracellular to extracellular environments. Its expression has been shown to result in a reduction in cell iron content (Zhang et al., 2024). FTH1 is the principal subunit of ferritin, the primary intracellular iron storage protein, which plays a pivotal role in regulating cellular iron homeostasis (Muhoberac and Vidal, 2019). GCLC represents the initial rate-limiting enzyme of GSH synthesis, functioning as the primary regulator of the cellular antioxidant system (An et al., 2024). Therefore, the genes in question act to either reduce cellular iron content or to facilitate GSH synthesis. This is an essential mechanism that prevents cells from undergoing ferroptosis and maintains them in a normal state. The downregulation of these genes may result in the ferroptosis of cells treated with Cd. Subsequently, quantitative reverse transcription polymerase chain reaction (qRT-PCR) was conducted on hepatic tissues, yielding results that were consistent with those obtained from RNA sequencing (Figure 2C). It is established that ferroptosis is principally initiated by the accumulation of deleterious lipids, particularly lipid hydroperoxides, in which malondialdehyde (MDA) represents the ultimate product of lipid peroxidation reactions. It was observed that treatment with Cd resulted in a significant elevation in the serum level of the lipid oxidation end product MDA, whereas this trend was reversed by cotreatment with Se (Figure 2E). Furthermore, GSH is the most prevalent antioxidant within cells, whereas GSSG represents its oxidized form. A negative correlation has been observed between GSH/GSSG levels and ferroptosis. Similarly, the ratio of reduced to oxidized glutathione was decreased by Cd treatment and increased by Se supplementation (Figure 2F), indicating that ferroptosis was induced by Cd and prevented by Se.

To further test these hypotheses, we conducted a detailed examination of mitochondrial morphology using electron microscopy. In comparison to the control group, the mitochondria in the Cd-treatment group exhibited wrinkled and reduced morphology, accompanied by increased electron density in the bilayer membrane and disorganized mitochondrial cristae structure (Figure 2G). These mitochondrial morphological alterations—including reduced organelle size, increased membrane density, and cristae disruption—are established ultrastructural hallmarks of ferroptosis and are functionally associated with impaired mitochondrial bioenergetics, including loss of membrane potential, diminished electron transport chain activity, and excessive ROS generation (Dixon et al., 2012). In contrast, the mitochondria in the group treated with a combination of Se and Cd exhibited a relatively normal mitochondrial structure (Figure 2G). Furthermore, Se supplementation alone did not result in any alteration to the liver mitochondrial structure (Figure 2G). We further performed the mouse hepatic organoid, similarly, the growth of hepatic organoid was significantly inhibited by Cd treatment, whereas the administration of Se was observed to effectively reverse the growth impairment induced by Cd exposure (Figure 2H).

### 3.3 Severe ferroptosis is observed in Cd-exposed kidney tissue

**FIGURE 3:**
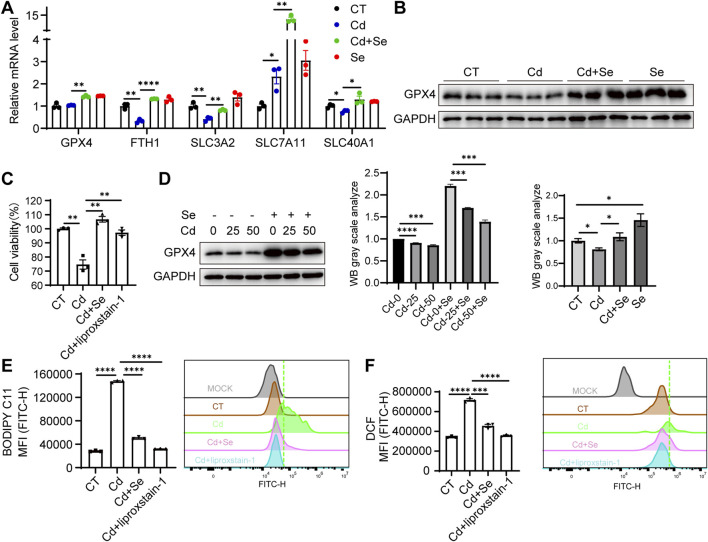
Severe ferroptosis is observed in Cd-exposed kidney tissue. **(A)** Expression of anti-ferroptosis genes in mouse kidney by reverse transcription-polymerase chain reaction (RT-PCR). Values were normalized to the mRNA level of the housekeeping gene Actin in the respective sample. **(B)** Immunoblotting of GPX4 and GAPDH in mouse kidney. The figure below shows the quantitative analysis of GPX4 protein expression. **(C)** CCK8 assay for renal HK2 cell survival under SeMet, CdCl_2_ and ferroptosis inhibitor liproxstain-1 treatment. **(D)** Immunoblotting for detection of GPX4 in SeMet and CdCl_2_-treated HK2 cells. The right panel shows the quantitative analysis of GPX4 protein expression. **(E–F)** Detection of BODIPY C11 **(E)** and **(F)** ROS level of HK2 cell under different treatments. Data are expressed as mean ± SEM. Pairwise comparisons between groups were conducted using two-tailed non-paired Student’s t tests. ns, not significant (p ≥ 0.05); *p < 0.05; **p < 0.01; ***p < 0.001.

Cd treatment caused pronounced ferroptosis to occur in liver tissue, and the myeloperoxidase (MPO) features showed even more severe damage in the kidney (Figure 1I). We then next assessed whether the mechanism of ferroptosis involved in process of the kidney injury. Similarly, the expression of genes associated with resistance to ferroptosis was significantly reduced in the kidneys of mice exposed to Cd, and renal GPX4 was significantly diminished (Figures 3A,B). These ferroptosis phenomena were reversed by Se supplementation (Figures 3A,B). This finding was also corroborated in the renal HK2 cell line. Cd treatment resulted in a reduction in the survival of HK2 cells (Figure 3C). Subsequent assays demonstrated that Cd diminished GPX4 levels in HK2 cells and augmented BODIPY C11 and ROS levels, which are indicative of ferroptosis (Figures 3D–F). Furthermore, Se supplementation in Cd-treated HK2 cells resulted in increased GPX4 levels and the reversal of a range of Cd-induced ferroptosis phenotypes (Figures 3C–F), which was consistent with the effect exerted by the ferroptosis inhibitor liproxstain-1 (Figures 3C–F).

In our study, we confirmed that the kidney is a primary target of Cd toxicity, and Cd exposure induced ferroptosis in renal tissue. Notably, we observed a significant upregulation of SLC7A11 following Cd treatment. SLC7A11, as the light-chain subunit of the cystine/glutamate antiporter system Xc^−^, plays a critical role in ferroptosis resistance. It facilitates the import of extracellular cystine, which is subsequently reduced intracellularly to cysteine and utilized for GSH synthesis. GSH serves as an essential cofactor for GPX4, an enzyme that detoxifies lipid hydroperoxides and prevents the accumulation of harmful lipid ROS. Through this pathway, SLC7A11 contributes to the maintenance of redox homeostasis and protects cells from undergoing ferroptotic death (Dixon et al., 2012; Yang et al., 2014). Interestingly, we found that the upregulation of SLC7A11 was more pronounced in the liver than in the kidney, suggesting a potentially stronger ferroptosis resistance in hepatic tissue (Figures 2C, 3A). This disparity may partly explain why cadmium-induced injury was more severe in the kidney compared to the liver.

### 3.4 Cd-induced hepatorenal communication exacerbates renal ferroptosis via exosomes

**FIGURE 4:**
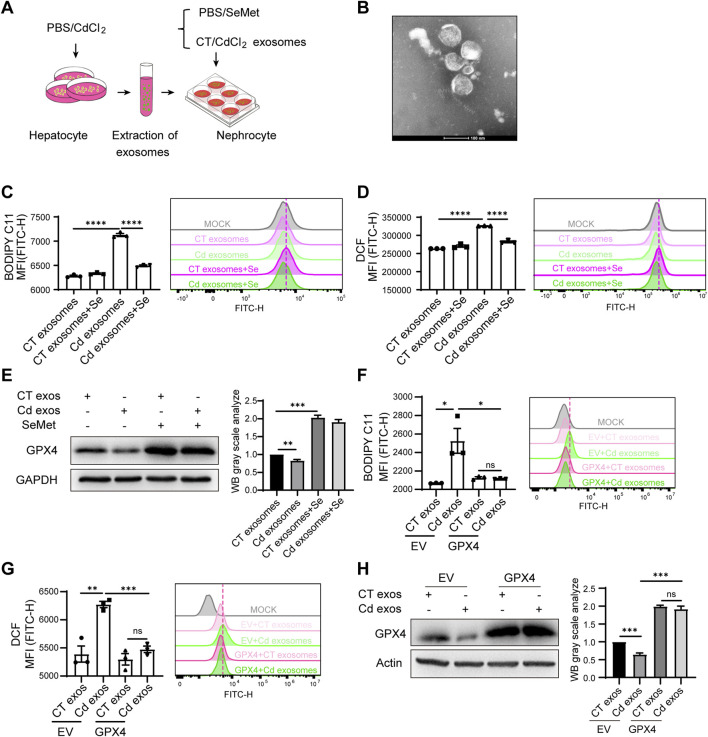
Cd-induced hepatorenal communication exacerbates renal ferroptosis via exosomes. **(A)** Pattern diagram: liver primary cells were isolated from mice and treated with PBS/CdCl_2_ for 24 h. Cell supernatants were collected and exosomes were extracted. HK2 cells were treated with CT/CdCl_2_ exosomes in combination with SeMet. **(B)** Representative images of the morphology of cellular supernatant exosomes observed by transmission electron microscopy. Scale: 100 μm. **(C)** Detection of BODIPY C11 and **(D)** ROS level of HK2 cells. HK2 cells were treated with CT/CdCl_2_ exosomes and SeMet for 24 h. **(E)** Immunoblotting for detection of GPX4 and GAPDH in CT/CdCl_2_ exosomes and SeMet HK2 cells. The right figure shows the quantitative analysis of GPX4 protein expression. **(F)** Detection of BODIPY C11 and **(G)** ROS level of HK2 cells. HK2 cells were transfected with either empty vector (EV) or a GPX4-overexpressing plasmid, followed by treatment with exosomes isolated from the supernatant of CT/CdCl_2_ treated Huh7 cells. **(H)** Western blot analysis of GPX4 and Actin expression in HK2 cells. The processing conditions are in accordance with those of **(F)** and **(G)**. The right figure shows the quantitative analysis of GPX4 protein expression. Data are expressed as mean ± SEM. Pairwise comparisons between groups were conducted using two-tailed non-paired Student’s t tests. ns, not significant (p ≥ 0.05); *p < 0.05; **p < 0.01; ***p < 0.001.

Thus, we next explored whether the liver contributes to kidney damage during Cd exposure. Exosomes are known to facilitate the transfer of information between cells, even over long distances. This raises the question of whether liver-derived exosomes may play a role in targeting the kidney and modulating kidney injury. To this end, we initially investigated the impact of liver cell-derived exosomes on kidney cells. We employed the hepatocyte-related cell line Huh7, which has a high exosome production rate and is easy to handle, and treated it with Cd for 24 h. The cell supernatants were then collected and the exosomes were isolated using ultracentrifugation (Figures 4A,B). The treatment of renal HK2 cells with exosomes derived from Cd-treated liver cells resulted in the unexpected observation of a heightened capacity to induce ferroptosis in kidney cells, as evidenced by elevated lipid peroxidation and ROS levels (Figures 4C,D). Consequently, the ferroptosis-resistant factor GPX4 was found to be downregulated, indicating that liver exosomes treated with Cd are capable of inducing ferroptosis in the kidney (Figure 4E). Furthermore, Se supplementation effectively reversed this process, not only rescuing GPX4 levels in HK2 cells, but also alleviating the Cd-treated liver exosomes-induced ferroptosis (Figures 4C–E). By expressing GPX4 in the renal cells, we observed that GPX4 overexpression diminished the renal ferroptosis exerted by exosome released from Cd-exposed hepatocytes, supporting the notion that GPX4 is the master regulator from Cd-exosome induced renal ferroptosis (Figures 4F–H).

Thus, we identified a new mechanism of hepatorenal communication in Cd toxicity to the organ, where Cd-induced hepatic exosome exacerbates renal ferroptosis.

### 3.5 Antagonistic effect of Se on Cd-induced ferroptosis via upregulation of GPX4

**FIGURE 5:**
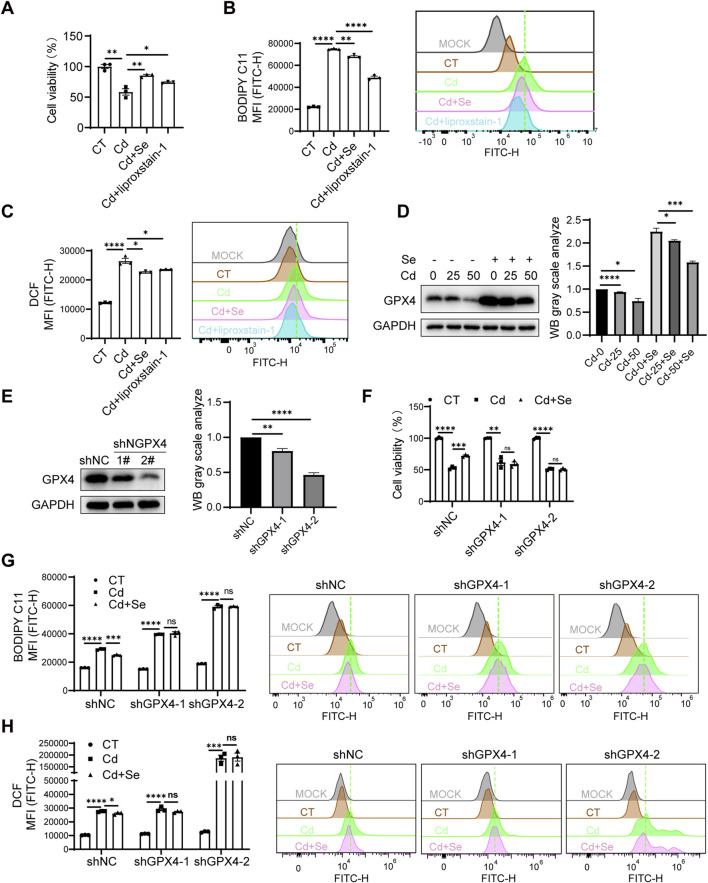
Antagonistic effect of Se on Cd-induced ferroptosis via upregulation of GPX4. **(A)** CCK8 assay for AML12 cell survival under SeMet, CdCl_2_ and ferroptosis inhibitor liproxstain-1 treatment. **(B)** Detection of BODIPY C11 and **(C)** ROS level of AML12 cell under different treatments. **(D)** Immunoblotting for detection of GPX4 in SeMet and CdCl_2_-treated AML12 cells. The right panel shows the quantitative analysis of GPX4 protein expression. **(E)** Immunoblotting for detection of GPX4 knockdown efficiency in AML12 cells. The right panel shows the quantitative analysis of GPX4 protein expression. **(F)** CCK8 assay cell survival of cell lines with AML12 knockdown of GPX4 after cadmium and/or selenium treatment. **(G)** Detection of BODIPY C11 and **(H)** ROS level of cell lines with AML12 knockdown of GPX4 after cadmium and/or selenium treatment. Data are expressed as mean ± SEM. Pairwise comparisons between groups were conducted using two-tailed non-paired Student’s t tests. ns, not significant (p ≥ 0.05); *p < 0.05; **p < 0.01; ***p < 0.001.

To further investigate the protective mechanism of Se supplementation against Cd-induced organ ferroptosis, we first validated this phenotype in AML12 cells. Indeed, the CCK8 assay revealed that Cd induced AML12 cell death, whereas Se alleviated the growth-inhibitory effect of Cd (Figure 5A). To further ascertain the ferroptosis status of the cells, we employed BODIPY-C11 and DCFH-DA fluorescence probes to quantify the levels of lipid peroxidation and ROS individually, both of which are hallmark indicators of cellular ferroptosis. The results demonstrated that Cd markedly enhanced the ferroptosis of AML12 cells, and this effect was markedly diminished by Se supplementation (Figures 5B,C). Notably, the rescue effects of Se on cell survival and ferroptosis markers were almost identical to those of the ferroptosis inhibitor liproxstatin-1 (Figures 5A–C), which suggests that Se plays a pivotal role in ferroptosis inhibition during Cd treatment in cells. These results suggested that we were able to replicate this phenotype in AML12 cells, i.e., Se supplementation was able to alleviate Cd-induced ferroptosis.

It was established that ferroptosis is caused by iron overload or the accumulation of ROS, which leads to lipid peroxidation, the key mechanism underlying the onset of ferroptosis (Endale et al., 2023). GPX4 is a pivotal antioxidant enzyme that eliminates ROS and prevents cells from undergoing ferroptosis. This enzyme functions in a Se-dependent manner, utilizing GSH to catalyze the reduction of lipid peroxides. This indicates that Se supplementation may mitigate Cd toxicity by enhancing GPX4’s capacity to mitigate hepatic ferroptosis. We therefore proposed that Se supplementation may antagonize the mechanism of Cd toxicity by upregulating GPX4 expression. This hypothesis was initially corroborated by the observation that GPX4 exhibited a marked decline in expression in Cd-exposed livers, whereas Se supplementation was found to restore its expression (Figure 2D). Subsequently, Cd treatment resulted in a significant reduction in the protein level of GPX4 in AML12 cells, with a dose-dependent effect. Conversely, Se supplementation was observed to elevate GPX4 expression (Figure 5D), suggesting GPX4 mediates the ferroptosis process initiated by Cd and Se treatment.

To confirm whether GPX4 is involved in the mechanism by which Se protects Cd-induced ferroptosis, we generated AML12 stable cell lines in which GPX4 expression was knocked down (Figure 5E). The results demonstrated that GPX4 knockdown exacerbated the lipid peroxidation and ROS production of AML12 cells induced by Cd treatment (Figures 5G,H). Furthermore, the knockdown of GPX4 negated the protective effect of Se against Cd-induced ferroptosis and cell survival impairment (Figures 5F–H), indicating the necessary role of GPX4 in Se-induced rescue effect. In light of these findings, we can conclude that Cd exposure may induce ferroptosis through GPX4 inhibition, and Se supplementation antagonize hepatic ferroptosis, largely through the upregulation of GPX4.

Notably, we found that knockdown of GPX4 in renal HK2 cells would result in the failure of HK2 cells to survive after the knockdown of GPX4, suggesting the importance of GPX4 to the kidney. It also explains the sensitivity of reduced GPX4 levels to the damage of renal ferroptosis.

### 3.6 Cd-induced liver exosomal miR-2137 mediates renal cell ferroptosis and is blunted with Se

**FIGURE 6:**
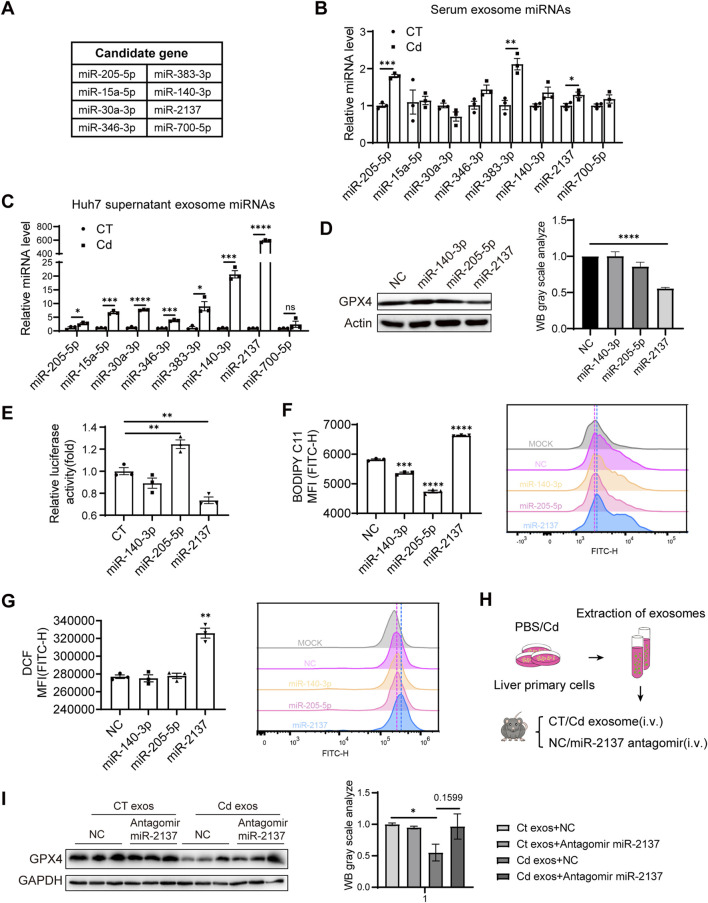
Cd-induced liver exosomal miR-2137 mediates renal cell ferroptosis and is blunted with Se. **(A)** Prediction of miRNAs targeting GPX4 using TargetScan. **(B)** Detection of serum levels of miRNAs targeting predicted miRNAs in control and CdCl_2_-exposed mice. **(C)** Detection of levels of miRNAs targeting predicted miRNAs in supernatant exosomes of PBS/CdCl_2_-treated Huh7 cells. **(D)** GPX4 and Actin levels were detected by immunoblotting after transfection of 293T for 48 h using miRNAs. The right panel shows the quantitative analysis of GPX4 protein expression. **(E)** Regulation of GPX4 3‘UTR by miRNAs was detected using dual luciferase in 293T cells. **(F)** Detection of BODIPY C11 and **(G)** ROS level in HK2 cells transfected with different miRNAs. **(H)** Pattern diagram: liver primary cells were treated with PBS/CdCl_2_ for 24 h, supernatants were collected and exosomes were isolated. Exosomes were injected into mice by tail vein and NC/miR-2137 antagomir treatment was given twice a week for a total of 2 weeks. **(I)** Immunoblotting to detect the expression levels of GPX4 and GAPDH in mouse kidney. The right panel shows the quantitative analysis of GPX4 protein expression. Data are expressed as mean ± SEM. Pairwise comparisons between groups were conducted using two-tailed non-paired Student’s t tests. ns, not significant (p ≥ 0.05); *p < 0.05; **p < 0.01; ***p < 0.001.

Next, we explored the mechanism of Cd-induced downregulation of renal GPX4 by hepatic exosomes. In our murine model, exposure to Cd resulted in unaltered GPX4 mRNA expression but a reduction in protein level (Figures 3A,B), suggesting the involvement of miRNAs in GPX4 downregulation. Given the abundance of miRNA content in exosomes, we conducted an online prediction of potential miRNAs targeting the mouse GPX4 mRNA 3′UTR using the TargetScanMouse 8.0 tool, resulting in the identification of eight miRNAs conserved between humans and mice (Figure 6A). The serum exosomes of the mice model exhibited elevated levels of miR-205-5p, miR-383-3p, and miR-2137 in response to Cd exposure (Figure 6B). The detail sequences for miRNA quantitative RT-PCR are listed in Table 1 and 2, respectively. In the Huh7 cells, Cd treatment resulted in the upregulation of seven miRNAs in the exosomes (Figure 6C). Consequently, miR-140-3p, miR-205-5p and miR-2137 were selected for the subsequent assay. Following the transfection of these miRNA mimics into 293T cells, it was observed that only miR-2137 resulted in a notable reduction in GPX4 expression (Figure 6D). Furthermore, only miR-2137 was observed to repress the fire luciferase (FL) levels present in the 3′UTR of mouse GPX4 mRNA (Figure 6E), thereby indicating that GPX4 is the direct target gene of miR-2137. Indeed, among these miRNAs, transfection of miR-2137 resulted in an increase in the production of the ferroptosis marker lipid peroxidation and ROS in HK2 cells, while transfection of miR-140-3p or miR-205-5p exhibited an inhibitory effect (Figures 6F,G). This is consistent with the GPX4 expression trend regulated by these miRNAs (Figure 6D), which suggests that GPX4 plays a master role in ferroptosis regulation. Therefore, the results above suggest that elevated miR-2137 may target the kidneys and repress GPX4 expression, thereby exacerbating kidney ferroptosis and injury.

To ascertain the significance of elevated exosomal miR-2137 in kidney ferroptosis in the context of Cd exposure, we isolated exosomes from the supernatant of Cd-treated liver primary cells and subsequently administered them to mice in conjunction with an miR-2137 antagomir via the tail vein at 2-week intervals for a period of 2 weeks (Figure 6H). Immunoblotting analysis of kidney tissues from mice revealed that the tail vein injection of Cd-treated liver cell exosomes significantly downregulated renal GPX4 levels compared to the control exosomes (Figure 6I). Notably, inhibition of miR-2137 was able to reverse the reduction in GPX4 expression observed in mice treated with Cd-treated liver cell exosomes. Although the densitometric quantification did not show statistical significance, an increasing trend in GPX4 protein levels was observed (Figure 6I). This suggests that the elevated levels of miR-2137 in these exosomes are responsible for the decreased GPX4 expression in the kidneys. It was observed that the inhibition of miR-2137 did not result in a change in GPX4 expression in mice treated with control exosomes (Figure 6I), indicating a lower level of miR-2137 in the control exosomes. Therefore, Cd functions as a stimulant, inducing the secretion of liver cell-derived exosomes with a high concentration of miR-2137. This, in turn, results in the inhibition of kidney GPX4 expression, thereby exacerbating renal ferroptosis. The upregulation of the GPX4 pathway by Se supplementation or miR-2137 block represents a promising therapeutic approach to alleviate the Cd-induced ferroptosis and tissue injury.

## 4 Discussion

This study demonstrates that both the hepatic and renal injury induced by Cd is linked to ferroptosis through the targeting of GPX4 in a Cd-exposed mice model. Specifically, we identified a novel mechanism of organ communication between the liver and kidneys, whereby renal cells are more sensitive to Cd exposure *in vivo*. The results of the mechanistic studies indicated that Cd induced the release of hepatocyte exosomes, which were found to be rich in a variety of miRNAs produced by hepatocytes. These included miR-2137 and others, which were demonstrated to be capable of targeting renal GPX4 and reducing the level of GPX4. This, in turn, was shown to induce the occurrence of ferroptosis and more severe damage in the kidneys. Specifically, the exosomes from Cd-exposed hepatocytes were sufficient to decrease the GPX4 expression of kidney tissue and the administration of miR-2137 antagomir effective in rescuing GPX4 expression, highlighting the importance of hepatocyte exosomal miR-2137 in reducing renal GPX4 expression and aggerating kidney injury. Furthermore, our findings indicate that Se supplementation can effectively mitigate hepatic exosome-induced renal ferroptosis by enhancing GPX4 signal. Therefore, our findings indicate the necessity for Se supplementation to protect Se detoxification and suppress Cd-induced ferroptosis via the promotion of selenoprotein GPX4 in the liver and kidney. Furthermore, our findings indicate the potential utility of exosomes as biomarkers and therapeutic agents for the treatment of Cd toxicity.

Ferroptosis is the recently identified form of regulated cell death, which is induced by iron-dependent lipid peroxidation. Given the established link between the toxicity of heavy metals, such as Cd, and iron metabolism, as well as the induction of oxidative stress, prior research has indicated the existence of a mutual interaction between metal toxicity and ferroptosis. In accordance with prior research, we observed liver and kidney injury in mice exposed to Cd (Figures 1B–I), which is linked to ferroptosis by downregulating GPX4 (Figures 2D, 3B). Furthermore, our findings revealed that the release of Cd-induced hepatocyte exosomes containing higher miR-2137 (Figure 6C), can target GPX4 (Figure 6E), thereby reducing GPX4 expression in renal cells and inducing additional ferroptosis (Figures 6D,F,G). Our findings indicated that the release of liver exosomes represent an important avenue by which some dysregulated miRNAs may target antioxidative factors in distal kidney and thus aggerate kidney ferroptosis.

The physiological mechanisms and detoxification processes associated with Cd poisoning have been the subject of considerable research interest. The kidney is the primary target organ for Cd exposure (Qing et al., 2021). Cd can be reabsorbed by the proximal tubule following its entry into the kidney via the bloodstream (Satarug et al., 2022). The proximal tubule represents the primary site of reabsorption, with the majority of glucose, amino acids, vitamins, and trace proteins present in the original urine undergoing reabsorption within this structure. These cells exhibit a greater number of mitochondria and a higher metabolic rate than other epithelial cells. Furthermore, Cd deposited in the cells is stored in the cytoplasm and mitochondria, which results in an alteration of the redox balance and the subsequent production of ROS (Bautista et al., 2024). Consequently, the kidneys may exhibit heightened sensitivity to Cd exposure. A number of studies have now demonstrated a correlation between Cd toxicity and ferroptosis. Cd exposure has been demonstrated to induce ferroptosis in mouse renal tubular epithelial cells via endoplasmic reticulum stress and autophagy (Zhao et al., 2021). The exposure of mice to environmentally relevant concentrations of Cd has been demonstrated to induce oxidative stress and excessive mitochondrial autophagy in the kidney (Qiu et al., 2023). Nevertheless, previous studies have concentrated on the immediate effects of Cd on the kidney. Our study utilizing mass spectrometry revealed that, despite the lower accumulation of Cd in the kidney compared to the liver (Figure 4H), exposure to Cd resulted in more pronounced kidney damage, as indicated by the detection of MPO (Figure 4G). This prompted us to consider the possibility of alternative mechanisms of action, beyond direct organ damage caused by Cd. The elevated liver exosomal miR-2137 (Figure 6C) suggests that miR-2137 may be originally upregulated in the liver cells that have suffered from Cd exposure. In the kidney of mice exposed to Cd, GPX4 mRNA levels remained unaltered, yet protein expression was diminished (Figures 4A,B), indicating miRNA-mediated downregulation of GPX4 expression may occur in kidney. Our data displayed that the liver exosomes suffered from Cd were sufficient to inhibit renal GPX4 expression *in vivo* (Figure 6I). Importantly, miR-2137 block by antagomir clearly restored GPX4 production (Figure 6I), showing the importance of miR-2137-containing liver exosomes in the control of GPX4 expression and ferroptosis in distal kidney. Renal GPX4 expression may be dictated by Cd with different manner, however, liver-originated exosomes exhibited considerable ability to repress renal GPX4 expression (Figure 6I). Moreover, we could not exclude the possibility that Cd reduced the GPX4 expression in the local liver and kidney, by upregulating miR-2137 in the local cells. Indeed, In the liver of mice exposed to Cd, GPX4 mRNA levels remained unchanged, but the protein expression was diminished (Figures 2C,D).

The antioxidant properties of Se have been the subject of extensive research (Li et al., 2023). The antagonistic effects of Se on heavy metals have been the subject of numerous studies, yet the precise mechanism of action remains unclear. Previous studies have shown that Se treatment effectively antagonized Cd-induced oxidative stress in the liver during Cd exposure (Jihen el et al., 2009). Furthermore, another study demonstrated that Se supplementation mitigated Cd-induced hepatocyte injury by enhancing the antioxidant system and reducing excessive autophagy and apoptosis (Zhang et al., 2017). The consumption of Se-enriched rice in mice has been demonstrated to provide protection against damage in the low Cd state of the body. Furthermore, it has been shown to support the enzymatic antioxidant system by eliminating oxidative damage (Su et al., 2021). Nevertheless, the precise mechanism through which Se exerts its effects on Cd-induced ferroptosis in hepatocytes remains unclear. The present study observed that Se supplementation effectively upregulated the protein levels of GPX4 in liver and kidney which was repressed by Cd exposure (Figures 2D, 3B). Furthermore, we observed that it exerted a beneficial effect on the reduction of GPX4 in the renal cells resulting from liver-released exosomes in the context of Cd exposure (Figure 4E). Notably, our results showed that Se, in contrast to Cd, inhibited serum exosomal miR-2137 levels in our mouse model (data not shown). This suggests that decreased exosomal miR-2137 may contribute to increased GPX4 expression in the kidney upon Se treatment (Figure 3B). It is also possible that Se downregulates miR-2137 in the local liver and kidney, thus protecting cells from ferroptosis caused by Cd exposure. Indeed, Se treatment resulted in a 1.5-fold increase in GPX4 mRNA and a 1.2- to 1.5-fold increase in protein levels in the liver and kidney, respectively. (Figures 2C,D, 3A,B). These results support the hypothesis that miRNA-mediated upregulation of GPX4 expression occurs under Se conditions through repressing miR-2137, thus protecting tissue injury comes from Cd upon local tissue damage and exosome avenue. Therefore, it can be speculated that Cd and Se regulate the expression of miR-2137 and its downstream target GPX4 in an opposite manner conducive or detrimental to ferroptosis in the liver and kidney cells.

Our findings have significant implications for the prevention of Cd-induced harm to the body. This is the first study to reveal the role of hepatorenal communication in organ ferroptosis due to Cd exposure. Previous studies on the mechanism of organ toxicity due to Cd exposure have been relatively homogeneous, with a notable absence of consideration given to the role of organ communication. The findings of our study offer new insights into the detoxification mechanisms underlying Cd poisoning. Additionally, we elucidate the protective mechanism of Se in organ ferroptosis due to Cd exposure, thereby providing evidence that Se antagonizes Cd toxicity.

The current study is not without limitations. The findings of our study indicated that exposure to Cd resulted in a reduction in GPX4 levels within the tissues. Nevertheless, the other signal pathways that may mediate the reduction of GPX4-induced tissue ferroptosis by Cd remain to be elucidated. A number of miRNAs were found to be upregulated in exosomes secreted by Cd-treated liver progenitor cells. For the purposes of this study, miR-2137, which was identified as the most effective in targeting GPX4 and was demonstrated the top upregulated in the serum exosomes of Cd-treated mice, was selected for further investigation. Currently, studies on miR-2137 remain limited. Existing research indicates that miR-2137 is a stress-inducible microRNA whose expression is regulated by the RNase activity of IRE1 and is significantly upregulated under conditions of endoplasmic reticulum stress, thereby promoting anabolic metabolism and cell growth (Hamid et al., 2020). In addition, miR-2137 is involved in immune responses and pro-inflammatory signaling, as observed in both macrophages and neuronal cells (McIlwraith and Belsham, 2023). These findings suggest that miR-2137 plays an important role in stress adaptation and inflammation regulation. However, whether miR-2137 also influences these physiological processes during cadmium exposure remains an important direction for future investigation. It is possible that other miRNAs may also play a role in regulating GPX4 in the human body. For example, miR-423-3p also exhibited a slight downregulation of GPX4 and was observed to upregulate lipid peroxidation and ROS levels in renal cells, thereby causing ferroptosis (data not shown). It can therefore be surmised that the downregulation of renal GPX4 due to hepatorenal communication during Cd exposure is the result of the combined effects of multiple exosomes. In addition, we found that miR-205-5p mimic did not decrease, but increase the luciferase activity of the 3′-UTR clone of mouse GPX4 mRNA. We proposed that GPX4 is not the *bona fide* target gene of miR-205-5p. Rather, miR-205-5p acts to upregulate GPX4 expression through an indirect mechanism. Furthermore, *in vivo* experiments in mice demonstrated that miR-2137 blockade also protected GPX4 in the kidney. The combination of Se and miR-2137 blockers may have a superior detoxification effect. Consequently, further research is required to elucidate the protective impact of Se on organ ferroptosis resulting from Cd exposure. Here, we did not screen the components other than miRNAs involved in renal injury exerted by liver-released exosomes in the context of Cd exposure. We considered that the exosomal proteins or lipids, may also have a role in liver-renal injury. We will continue to screen the exosomal components, to find the novel factor involved in renal toxicity. Our acute Cd exposure model must have certain difference with the chronic Cd exposure in the human population. However, both liver and kidneys are the most important targets for acute and chronic Cd exposure. In addition, overload oxidative stress and lipid peroxidation are their common initial mechanisms by which they induce organ damage. Our results may partially recapitulate the effect of chronic Cd exposure seen in the human, and our results may also have the suggestive significance to understand the chronic Cd exposure-induced renal injury. The exosomal miR-2137 induced renal injury needs to be further demonstrated in the chronic model. Selenium participates in various cellular processes through multiple selenoproteins. Therefore, further studies are needed to determine their specific roles in cadmium-induced ferroptotic responses. Since the clinical samples are unavailable currently, it is unclear whether the increased serum exosomal miR-2137 can also apply to the patients who exposed to Cd. Serum exosomal miR-2137 has the potential to be the biomarker for Cd exposure, which is a promising field that requires further investigation in the future.

**FIGURE 7:**
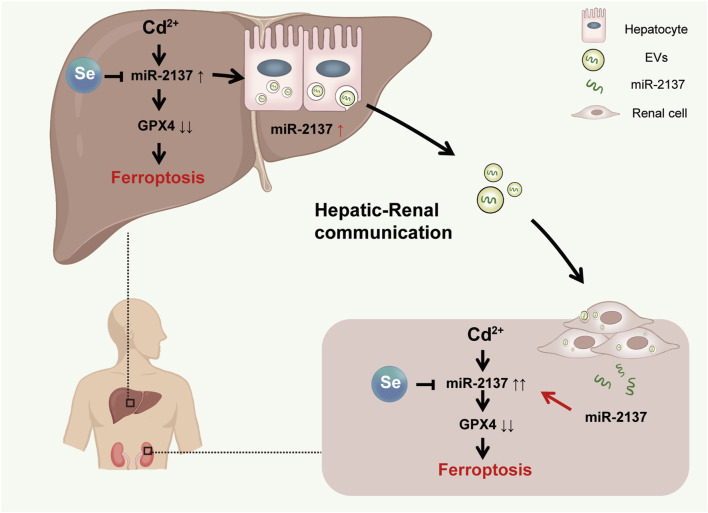
Model. Cd-induced renal ferroptosis by liver exosomes is rescued by Se supplementation. During Cd exposure, miR-2137 from hepatic exosomes targeted renal GPX4 and aggravated renal ferroptosis. Se supplementation alleviated renal ferroptosis exacerbated by hepatorenal communication by upregulating GPX4.

In conclusion, this study examined the intricate relationship between hepatorenal communication and Cd-induced organ ferroptosis, as well as the mechanism of Se detoxification in a Cd-exposed mouse model. From a mechanistic perspective, Cd-induced hepatic and renal ferroptosis can be attributed to the downregulation of the key anti-ferroptosis protein, GPX4. This occurs either directly or indirectly via organ exchange. The administration of Se was observed to mitigate the organ ferroptosis resulting from Cd exposure, as evidenced by the upregulation of GPX4 levels in the tissues. We have identified liver-derived exosomes and their implications for hepatorenal communication under conditions of Cd exposure (Figure 7). This provides a new target for further investigation into the mechanism of Cd-induced hepatorenal communication *in vivo*. Our findings indicate that exosomes may serve as biomarkers and potential therapeutic agents for the treatment of Cd toxicity.

## 5 Conclusion

Cd exposure has been demonstrated to induce tissue damage by decreasing the levels of GPX4 in mouse liver and kidney, thereby leading to ferroptosis. Concurrently, under conditions of Cd exposure, the liver targeted the renal GPX4 by secreting exosomes containing miR-2137, which in turn increased renal ferroptosis levels. The administration of Se was observed to mitigate the organ toxicity of Cd exposure to the liver and kidney, as evidenced by an increase in GPX4 levels.

## Data Availability

The RNA-seq data generated for this study have been deposited in the NCBI Gene Expression Omnibus (GEO) under accession number GSE302882 (https://www.ncbi.nlm.nih.gov/geo/query/acc.cgi?acc=GSE302882).
